# 

**Published:** 1815-08

**Authors:** 


### MONTHLY CATALOGUE OF MEDICAL BOOKS.

COMMENTARIES on some of the most Important Diseases of Children. By John Clarke, Esq. M.D. Part the First. Royal 8vo. Longman and Co.

Pharmacopœia Collegii Regalis Medicorum Londinensis, 1809. Editio Altera, 8vo. Longman and Co.

The Pharmacopœia of the Royal College of Physicians of London, 1809. Translated into English with Notes, &c. By Richard Powell, M.D. The Third Edition, 8vo. Longman and Co.

Minutes of Cases of Cancer and Cancerous Tendency, successfully treated by Mr. Samuel Young, Surgeon. 8vo. Cox and Son.

A Second Essay on Hydrocephalus Acutus, or Dropsy in the Brain. By J. Cheyne, M.D. 8vo. Underwood.

Outlines of the Physiognomical System of Drs. Gall and Spurzheim, indicating the Dispositions and Manifestations of the Mind. By J. G. Spurzheim, M.D. 12mo. Baldwin and Co.

A Critical Enquiry into the Pathology of Scrofula, in which the Origin of that Disease is accounted for on the New Principles. By George Henning, M.D. 8vo. Callow.

Elements of Medical Jurisprudence. By the late Samuel Farr, M.D. The Third Edition, corrected, and various Notes added by a Physician. To which are annexed, the late Dr. William Hunter's Observations on the uncertainty of the Signs of Murder in the Case of Bastard Children. 12mo. boards. Callow.

---

### *NOTICES TO CORRESPONDENTS.*

*We have, at length, received the American Journals, which contain many valuable papers. We were at first struck with the slow progress in some of the most hackneyed branches of surgery, till we recollected how often we have to make the same remark at home. A long case is given, which the writer calls Syphilitic, though it had none of the characters of that disease; and, after being exasperated by mercury, was cured by the* red oxyd of iron, *which, he was afterwards informed, Mr. Carmichael had applied nearly ten years ago in cancerous cases. The satisfaction derived from what the author calls an accordance of sentiment, will induce him, we doubt not, to suspect the character of the disease after perusing Mr. Carmichael's work on that subject; an account of which will be given in our next.*

*We are much obliged to Dr. Kinglake for the candid manner in which he has set the enquiry contained in page 97 of this Number afloat, and hope to receive the assistance of* Dr. Haxby, *and other Correspondents, on the subject. We have been for some time collecting Remarks on Contagion from several new publications which have been sent to us, and shall endeavour to concentrate and compare them together, to appear in a succeeding Number.*

*Communications have been received from Drs. Pinckard and Kinglake, Mr. Yeatman, N. W. &c. &c. &c.*

